# The acute management of acid assault burns: A pragmatic approach

**DOI:** 10.4103/0970-0358.63952

**Published:** 2010

**Authors:** A. Burd, K. Ahmed

**Affiliations:** Department of Surgery, Division of Plastic, Reconstructive and Aesthetic Surgery, The Chinese University of Hong Kong, Prince of Wales Hospital, Hong Kong

**Keywords:** Acute management, chemical assault, surgical timing

## Abstract

This case series comprises 31 patients who were victims of acid assault burns. They were admitted for acute or reconstructive care to a regional burns unit. Ten patients were admitted late with suboptimal acute care and needed a total of 50 reconstructive procedures. Of 13 patients admitted acutely, 7 had surgery performed after 48 hours of constant lavage while seven had urgent surgical debridement within 48 hours, followed by lavage. Although the number of reconstructive procedures performed in these two groups was similar, i.e., 20 and 19, respectively, the magnitude of the deformity in the urgent surgery group was significantly less than in the conventional surgery group. As in many cases of acute burns care, determining the evidence for best practice using a prospective, randomised, controlled comparison of conventional versus urgent surgery is difficult in view of the small number of cases involved. However, basing surgical practice on ethical principles, and in particular 'primum non nocere,' we propose that the urgent reduction of the chemical load on the skin by surgical debridement is appropriate in selected cases and should be considered in the acute management of these devastating injuries.

## INTRODUCTION

Acid assault burns are a particularly vicious form of attack where the motive is not to kill but to cause permanent disfigurement. The priority of the acute care is to limit the damage while the priority of the reconstructive care is to restore as much as possible the patient to optimum form and function. A recent review indicates that there are reports of such assaults that have occurred in many parts of the world but there appears to be a rising incidence in the developing countries where medical resources are limited.[1] Bangladesh has the highest reported incidence.[2] A worrying feature is that acid assaults are increasing in incidence at a time when the overall incidence of assaults is decreasing.[3] An indication of the perception of the problem, however, is that recent comprehensive reviews of acute burns management from authors in developed countries do not even mention such burns[4,5] although the significance of the problem was raised over 10 years ago.[6]

What is the evidence for the acute management of acid assault burns? Over the last 10 years, we have treated just 31 cases of acid assault burns in our unit. We have looked at the correlation between acute care and the reconstructive needs of the patients. This small case series does not provide high quality evidence but the problems of evidence based medicine in burns care have been discussed at both ends of the last decade[7,8] and the reality is that in many cases a randomised, prospective evaluation of treatments is not ethical. As such we proceed on a pragmatic basis and adhere to the principle ethical of '*primum non nocere*'. In this context, leaving acid on the skin of the assault victim should be regarded as '*nocere*'.

## PATIENTS AND METHODS

The burns unit at the Prince of Wales Hospital (PWH) is a regional referral centre covering a population of just under 4 million for acute major burns in Hong Kong. In addition, being close to the border with Mainland China, Hong Kong residents living in China return to PWH if they sustain major burns in the Mainland. In the last 10 years, we have admitted 31 cases of acid assault burn and they fall into four categories:

- delayed referrals with suboptimal acute care;
- acute referrals treated with no surgery;
- acute referrals treated with conventional surgery (after 48 hours); and
- acute referrals treated with urgent surgery (before 48 hours).

The conventional treatment consisted of immediate and continuous water lavage for 2–3 days, then excisional surgery and grafting within 1 week if necessary.

Our experience has been that even with early full thickness skin excision on 2^nd^ to 3^rd^ post burn day, there is a very high tendency for hypertrophic scarring to develop within skin grafts and particularly at graft junctions. This suggests that there is possibly an augmented healing response that may be secondary to a prolonged inflammatory stimulus. Thus, with intensive and continuous lavage and early excisional surgery we were still obtaining poor results. In view of this experience, we decided to change our acute management strategy.

Our current policy is outlined in Table 1.

**Table 1:** Our protocol for acute management of the acid assault burn

Determine extent and severity of injury on admission to the accident department
Commence immediate lavage with running water
Arrange for immediate eye consultation if there is eye involvement
For confluent areas of discoloured skin greater than 20 cm^2^ on face and 100 cm^2^ on the trunk or limbs, arrange for urgent examination under anaesthesia (EUA) in the operating theatre
For smaller burns arrange for transfer to burns unit and continue lavage
For patients undergoing an EUA, perform a test shave to determine the representative depth of injury and shave entire burn to achieve punctate bleeding
Continue lavage by applying wet dressing changed every 2 hours for 48 hours
At 48 hours, apply porcine skin to wound to test graft bed
24–48 hours later, return the patient to theatre for supplementary shave if necessary and definitive grafting with thick split thickness graft and over graft the donor site

Post-burn reconstruction is provided based on functional and aesthetic demands. Priority is always given to eye closure, oral continence, neck and limb movement. Surgery is also offered for resurfacing, hair transplantation and aesthetic nasal reconstruction. The reconstructive sequelae for each patient group have been categorised into minor or major. Minor procedures include simple grafts for eyelid contractures, hair transplants and z-plasties. Major procedures include free tissue transfer, tissue expansion and major facial resurfacing procedures.

## RESULTS

**Figure 1:**
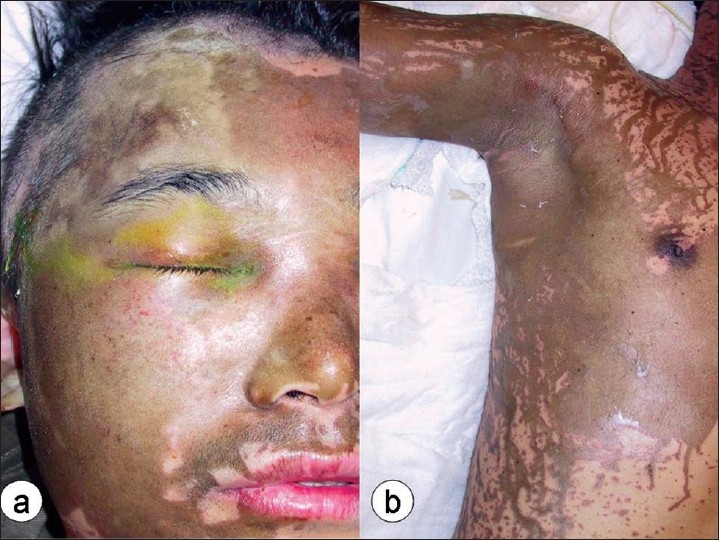
Confluent burn on the face (a) and limbs (b) that are indications for urgent surgical debridement

Figure 1 shows the appearance of confluent discolouration of the face and trunk, which would be an indication for an immediate examination under anaesthesia, test shave and surgical debridement.

**Figure 2:**
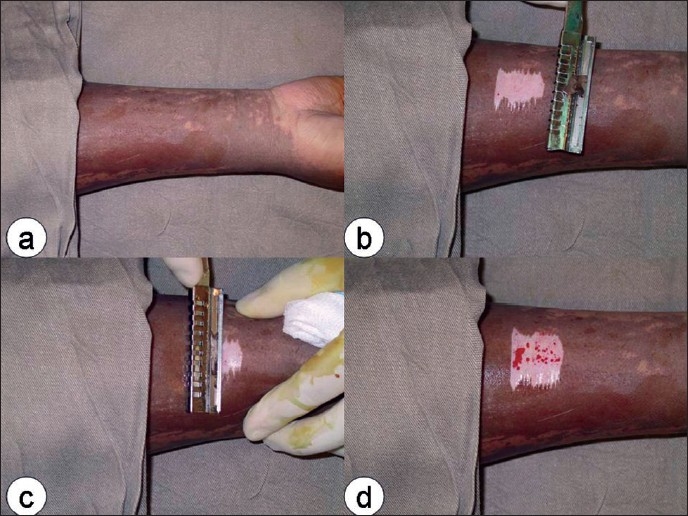
Tangential incision of an acid assault burn affecting the right forearm (a) showing no bleeding after the first (b) or second (c) shaves and some punctate bleeding (d) when reaching the lower dermis

Figure 2 shows the appearance of sequential shaves on a limb burn indicating significant depth of involvement at the very early stage post assault.

Table 2 shows the breakdown of the demographics, extent and distribution of the burn, management of the acute phase and the reconstructive sequelae. Of note, patient number 6 was the only patient who had no surgery in the acute phase but needed reconstruction later. This patient had acid splashed on her face, was beaten over the head with an iron bar and had both nipples cut off with scissors. She received a composite graft to reconstruct her left ala. She also had bilateral nipple areola reconstruction but this has not been included as a reconstructive sequela of the chemical assault.

**Table 2:** Details of the patient group demographics, extent and distribution of burn and management

	*Initials*	*Age (years)*	*Sex*	*%BSA*	*Distribution*	*Acute care*	*Reconstruction minor*	*Reconstruction major*
1	LYP	37	M	4.5	F	No surgery	0	0
2	CYL	29	F	0.2	F	No surgery	0	0
3	CPH	47	F	4.5	F, RUL	No surgery	0	0
4	LYK	29	M	2	AT, RUL	No surgery	0	0
5	WHC	43	F	1	F, RUL	No surgery	0	0
6	LLK	43	F	1	F	No surgery	1	0
7	TSM	38	F	1	F, AT	No surgery	0	0
8	KKC	43	F	7.5	F, RUL, LLL	Delayed	0	0
9	LCF	22	M	2	F, PT, RLL	Delayed	0	0
10	CYL	44	M	33	F, AT, LUL, LLL	Delayed	8	2
11	HYD	35	F	3.5	F, LUL	Delayed	3	1
12	LSM	28	F	25	F, AT	Delayed	0	1
13	TD	2	M	10	F, AT	Delayed	1	4
14	HSH	32	F	10	F, AT, RUL, LUL	Delayed	10	4
15	LKM	64	F	8	F, AT, RUL, LUL	Delayed	5	5
16	TWM	31	F	8	F, RUL, LUL	Delayed	3	3
17	SLH	48	F	50	PT, RUL, LUL, F	Delayed	0	0
18	LST	25	M	7	F, RUL, LUL	Conventional	0	0
19	NP	31	M	33	F, RUL, LUL, AT	Conventional	0	0
20	NSY	35	M	12.5	F, RUL, LUL, AT	Conventional	2	1
21	CKP	34	M	12	F, RUL, LUL	Conventional	8	2
22	TYF	44	F	6	F, AT	Conventional	0	0
23	HKH	44	F	10	F, RUL, LUL	Conventional	6	1
24	LOI	37	F	2	BUTTOCK	Conventional	0	0
25	LKW	60	M	15	F, AT, RUL, LUL	Urgent	0	0
26	HFC	27	M	10	F, RUL, LUL	Urgent	3	1
27	YTY	44	F	2.5	F, AT	Urgent	7	2
28	WMS	35	M	5.5	F, LUL, LLL	Urgent	6	0
29	PLY	77	M	13	F, AT, RUL, LUL	Urgent	0	0
30	SW	47	M	14	F, RUL, LUL	Urgent	0	0
31	LPY	42	M	7.5	F, LUL	Urgent	0	0

Conventional surgery has been defined in the text and refers to the general practice of continuous lavage for 48 hours and proceeding to excisional surgery within 1 week post burn. Delayed surgery indicates that the patient needed surgery but this has taken place several weeks after the burn injury [BSA - Burn surface area, F - Face, AT - Anterior trunk, PT - Posterior trunk, RUL - Rt. upper limb, LUL - Lt.uppet limb, RLL - Rt.lower limb, LLL - Lt.lower limb]

Patient number 16 was blinded in the assault and received a number of challenging reconstructive procedures. Tragically, she committed suicide 5 years post injury.[9] Patient 17 was the only mortality in the acute phase in this series. This patient had been assaulted by her co-workers in a restaurant and was admitted to a local hospital. She had been managed there for 10 days and had undergone surgical debridement but no grafting. She was transferred to our unit in a state of pseudomonas septicaemia and despite prolonged and intensive support failed to heal with repeated grafting and eventually succumbed 2 months post assault. Patient 19 illustrated a local relationship problem with the Hong Kong male having a Hong Kong wife and a Mainland mistress. When the wife discovered the infidelity, she threw acid on her husband as he showered and then committed suicide by leaping from their tower block apartment. With regard to the immediate treatment group, patients 26 and 28 were both males who requested aesthetic surgical procedures for eyelid and eyebrow reconstruction. Patient 27 was the only case of free flap failure in this series of patients. Of significance was that she had two free flaps that both failed with a similar pattern of anastomotic patency and no peripheral perfusion.

Table 3 summarises the results from the four groups.

**Table 3:** The reconstructive burden

	*Number*	*Reconstruction*		
		*Minor*	*Major*	*Total*
No surgery	7	1	0	1
Delayed surgery	10	30	20	50
Conventional surgery	7	16	4	20
Urgent surgery	7	16	3	19

Of note, one patient in the urgent surgery group had a failed free flap which was replaced with a further free flap. If this flap failure had not occurred, the reconstructive need for the urgent surgery group would have been significantly less in the major procedure category

## DISCUSSION

The results from this small case series indicate that when acute treatment is delayed, there is a significant need for reconstruction. What they do not show conclusively is that if the operation is done urgently to reduce the acid load, the reconstructive need is less than if the surgical removal of dead tissue is delayed for several days. Intervention has to be assessed by outcome but there is still considerable debate about how outcomes should be assessed. These difficulties have been reviewed in an excellent article by Falder *et al*[10] who reflect on the heterogeneity of the burns population. The majority of 'reconstructive' procedures performed in our urgent surgery group were performed on two male patients both of whom were probably victims of mistaken identity. Their demand for reconstruction was high. There were other patients in whom scarring, asymmetry and other deformities were objectively worse but they did not want further surgery.

We then return to the original question. What is the most appropriate management of the acute acid assault patient? We feel that it would be unethical to determine the evidence on the basis of a randomised, prospective, comparative clinical trial, when our sequential comparative case series indicates a better result with acute surgical reduction of the chemical load. The problem is that there are no statistical methods to demonstrate this with such a small and heterogenous group. So how do we manage the next case without the comfort and support of scientific evidence? We either follow the conventional teaching or proceed cautiously and ethically on the basis of *primum non nocere*. In this context, our contention is that the failure to rapidly remove chemicals that are causing permanent damage to the skin is an act of harm. One criticism of this approach is that we may be exposing a patient to unnecessary risks and complications of a skin graft in a wound that would have otherwise healed. The surgical technique, however, involves a 'test shave'. This is using the tangential excision technique but beginning with a thin excision primarily so as not to remove viable tissue. As data accumulate, statistical analysis will validate or negate our intuitive approach. For the next case though, we will be continuing with urgent examination under anaesthesia and progressing to acute surgical debridement as necessary.
